# Effect of concentrate supplementation on nutrient digestibility and growth of Brahman crossbred cattle fed a basal diet of grass and rice straw

**DOI:** 10.1186/s40781-015-0068-y

**Published:** 2015-09-25

**Authors:** Do Van Quang, Nguyen Xuan Ba, Peter T. Doyle, Dau Van Hai, Peter A. Lane, Aduli EO Malau-Aduli, Nguyen Huu Van, David Parsons

**Affiliations:** Institute of Animal Science Southern Vietnam, Ho Chi Minh, Vietnam; Faculty of Animal Sciences, Hue University of Agriculture and Forestry, Hue City, Vietnam; Peter Doyle Consulting, 4 Red Bean Close, Byron Bay, NSW 2481 Australia; Tasmanian Institute of Agriculture and School of Land and Food, University of Tasmania, Sandy Bay, 7001 Australia

**Keywords:** Bos indicus, Concentrate, Crossbred, Digestibility, Rice Straw, Vietnam, Yellow cattle

## Abstract

**Background:**

An experiment was conducted in Vietnam to test the hypothesis that total dry matter (DM) intake and liveweight (LW) gain would increase in a curvilinear manner with increasing amounts of concentrate offered.

**Method:**

There were five treatments: a basal diet of Guinea grass fed at 1 % of LW and rice straw fed *ad libitum* (T0), or this diet supplemented with concentrate at 0.6 (T1), 1.2 (T2), 1.8 (T3), or 2.4 % of LW (T4). The concentrate comprised locally available ingredients, namely cassava chips, rice bran, crushed rice grain, fishmeal, salt, and urea, mixed manually.

**Results:**

Concentrate intake increased from T0 to T3, but there was no difference in concentrate intake between T3 and T4. Total feed intake increased in a curvilinear manner from 4.0 to 6.4 kg DM/d as the quantity of concentrate consumed increased. The substitution of concentrate for grass and rice straw increased with increasing consumption of concentrate and was as high as 0.49 kg DM reduction per kg of concentrate consumed. LW gain increased curvilinearly, with significant differences between T0 (0.092 kg/d), T1 (0.58 kg/d) and T2 (0.79 kg/d); but there were no significant differences in LW gain between T2, T3 (0.83 kg/d) and T4 (0.94 kg/d).With increasing amount of concentrate in the diet, the digestibilities of dry matter, organic matter, crude protein, and crude fat increased, but NDF digestibility decreased.

**Conclusion:**

Based on these results, young Vietnamese Brahman-cross growing cattle will respond to a locally-sourced concentrate mix offered at a level of up to 1.2 % of LW.

## Background

Beef cattle production in Central Vietnam is concentrated in low-input and small-scale enterprises. Farmers generally have limited knowledge in terms of breed improvement, and feed and feeding management; hence, livestock production and enterprise productivity remain low. Little published information is available for the region; however there are some indicators of low performance. For example, a survey of cattle performance in Binh Dinh and Phu Yen provinces found that the average calving interval is longer than one year (476 days for Binh Dinh and 397 days for Phu Yen) [1]. For growing animals, basal diets of grass and straw can result in liveweight gains of 0.1 to 0.2 kg/day [2, 3]. Opportunities exist to improve feed efficiency and growth rate in cattle in Central Vietnam through effective supplement utilisation to enhance the intake of digestible energy and protein.

Many cattle in the region graze native grasses during the day and are often provided with crop products and by-products at night [4]. In general, native grass and rice straw can only meet the maintenance requirements of cattle, as they are low in metabolisable energy and protein [5, 6]. Ba et al. [7] showed that a range of introduced grasses can be productive in this environment, and a small but growing proportion of farmers confine cattle and feed basal diets of cut-and-carry native or sown grasses with by-products, such as rice straw, sugar cane tops, groundnut tops, and sweet potato leaves. While these crop by-products are useful sources of digestible and metabolisable energy for maintenance of cattle, they are often deficient in meeting the energy and protein requirements for growth in young beef cattle. Thus, some farmers provide locally available products (such as rice grain, rice bran, cassava leaves, and maize) that are not only highly digestible, but may also contain reasonable protein concentrations.

Supplementing Vietnamese cattle with energy-rich feeds and a source of protein can increase growth rates and reduce the time taken to attain market weight in finishing [2, 3]. However, the responses in liveweight (LW) gain to increasing amounts of supplementation vary depending on the composition of the concentrate and the interactions between the basal diet and supplement [8]. There are numerous reports in the published literature of substantial increases in liveweight gain (LWG) in cattle consuming low quality forages supplemented with energy and protein-rich feeds [9].

Diets and supplementary feed mixes for cattle in smallholder systems should be based on locally available forages, crop residues, and feed ingredients from agricultural by-products, because commercial complete mixed rations and feed supplements are in limited supply and are usually costly. A key challenge in Central Vietnam is to design diets and supplements that provide adequate metabolisable energy and protein for acceptable growth rates of young cattle. The objective of this research was to determine the effect of the amount of supplement (formulated from locally-available ingredients) on intake, nutrient digestibility, and growth of young cattle. We hypothesised that total DM intake and LWG would increase in a curvilinear manner with increasing amounts of concentrate offered.

## Methods

### Animals and experimental design

Twenty male cross-bred Brahman bulls of 11–12 months of age and weighing between 190–200 kg were fed at the Institute of Animal Sciences research station (Ben Cat District, Binh Duong Province) in Southern Vietnam. All experimental procedures were in accordance with the University of Tasmania Animal Ethics Committee guidelines, the 1993 Tasmania Animal Welfare Act and the 2004 Australian Code of Practice for the Care and Use of Animals for Scientific Purposes. Cattle were blocked on the basis of LW and allocated into 4 treatment groups of 5 animals per treatment. All animals were treated for internal and external parasites and vaccinated against foot and mouth disease, pasteurellosis, and rinderpest prior to the experiment. Cattle were kept in individual feeding stalls throughout adaptation and feeding periods. They were observed daily for any signs of discomfort caused by the housing, and daily feed intake was monitored and recorded.

The experimental design was a randomized complete block design with a control and four amounts of supplement: Control (T0) - basal diet of Guinea grass (fed at 1.0 % LW) and rice straw fed *ad libitum*; T1- the basal diet + concentrate fed at 0.6 % LW; T2: the basal diet + concentrate fed at 1.2 % LW; T3 - the basal diet + concentrate fed at 1.8 % LW; T4 - the basal diet + concentrate fed at 2.4 % LW.

The experiment had a duration of 98 days, comprising: an adaptation period of 14 days (26 Sep 2010 to 10 Oct 2010), a treatment period of 84 days (11 Oct 2010 to 11 Jan 2011), and a digestibility period of 7 days (the final 7 days of the treatment period).

### Feeds and their nutritive characteristics

Rice straw was purchased in one lot to minimise variation in characteristics throughout. It was dried, properly stored in a dry and well ventilated barn, chopped into 5-10-cm lengths and mixed well before feeding. Guinea grass was harvested at 30–40 days of re-growth, chopped into 5-10-cm lengths, and mixed well before feeding. Concentrate ingredients included cassava chips (34 % DM basis), rice bran (30 %), crushed rice grain (30 %), fishmeal (3 %), salt (1 %), and urea (2 %), which were manually mixed. The nutritive characteristics of the ration ingredients are shown in Table 1.

**Table 1:** Nutritive characteristics of the Guinea grass, rice, straw, and concentrate used in the experiment

Nutritive Characteristic	Guinea grass	Rice straw	Concentrate
Dry matter (%)	21.8	93.5	87.0
Ash (% DM)	5.3	17.5	7.5
Neutral detergent fibre (% DM)	72.5	73.6	11.4
Crude protein (% DM)	12.4	4.1	15.9
Ether extract (% DM)	1.6	1.1	5.0
Gross energy (MJ/kg DM)	19.5	15.8	18.2

### Feeding regime

The basal diet of Guinea grass was fed at 1.0 % LW in roughly two equal portions at 0800 and 1300 hours, with any residuals collected and weighed at 1800 hours. Rice straw was fed *ad libitum* once daily at 1830 hours, at 20 % above the previous day’s intake. The amount offered to each animal was adjusted once a week based on LW. Straw residues were collected at 0700 hrs prior to feeding grass each morning.

Mixed concentrate was offered to cattle twice daily in separate feeding troughs from the Guinea grass and rice straw. Concentrate was fed prior to offering the grass just before 0800 and at 1300 hours. Where the animals did not consume all of the concentrate within a short period after it was offered, they were allowed free access throughout the day. Residues were collected daily at 0700 hrs and weighed.

At the beginning of the adaptation period, all supplemented animals were fed a maximum of 0.5 kg of the mixed concentrate per day. The amount of concentrate was gradually increased by approximately 0.5 kg every second day up to the amount for the treatment. Each animal had free access to a 5-kg mineral block and water.

### Measurements

Cattle were weighed at 0630 hrs on two consecutive days at the start and end of the adaptation phase and weekly throughout the experimental period, to calculate daily LW change. The amounts of each feed offered and refused were weighed and sub-samples collected for dry matter determination daily. Additional sub-samples of each feed offered were collected daily, bulked within each 7 day period, and stored for analysis.

### Digestibility trial

During the last consecutive 7 days of the experimental period, total faecal output was manually collected for each animal. The output was thoroughly mixed each day and subsamples taken for DM determination (dried to a constant weight at 105 °C) and for laboratory analyses (about 5 % of the total). The subsamples for analyses were stored at −20 °C and bulked over the 7 days, after which they were defrosted, mixed, and further samples taken for nutrient composition analyses.

### Laboratory analysis

Dry matter of feeds, residues from individual animals, and faecal samples were determined by drying at 105 °C to a constant weight. Samples for chemical analysis were dried at 60 °C. Ash content was determined by heating samples in a furnace at 550 °C for 4 hours and organic matter (OM) content calculated as DM minus ash [10]. Neutral detergent fibre (NDF) was determined as described by Van Soest et al. [11]. Ether extract (EE) was determined using the standard Soxhlet fat extraction method [10]. Total nitrogen (N) was measured by the Kjeldahl procedure and crude protein (CP) calculated as N x 6.25. Gross energy (GE) of feed, residues and faeces was determined by bomb calorimetry (Bomb Calorimeter 6300, Parr Instrument Company).

### Calculations and statistical analyses

Liveweight gain was calculated from the difference between final and initial weights. Apparent digestibility of DM and OM, and digestibility of NDF were calculated as intake (kg DM/day) minus faecal output (kg DM/day) divided by intake (kg DM/day) expressed as a percentage. Substitution rate was calculated as the difference between control roughage (grass and rice strass) intake and treatment roughage intake, divided by concentrate intake.

Intake, LWG, and digestibility response variables were analysed in SAS (SAS Institute: The SAS system for Windows. v. 9.1. Cary, NC; 2003) [12] using PROC GLM with concentrate as a fixed effect, and a random block. Fisher’s protected LSD was used to test differences (*P* < 0.05) among means where the overall F test was significant. Regression equations were developed using the PROC GLM procedure, based on initial body weight and amount of concentrate offered and their quadratic terms as explanatory variables. Variables were dropped from the regression model if non-significant (*P* < 0.05) in the presence of other explanatory variables, and the regression re-calculated until only significant variables remained. The coefficient of determination (r^2^) and the overall F-test significance of the regression were determined. The regression equation is not presented where the overall F-test was not significant.

## Results

Summary statistics (mean, standard deviation, and range) for the key outputs are shown in Table 2. Table 3 contains regression results and Table 4 contains analysis of variance results.

**Table 2:** Summary statistics of feed intake, liveweight, average daily gain and organic matter digestibility by treatment group

	Treatment									
	0		0.6		1.2		1.8		2.4	
	Mean ± SD	Range	Mean ± SD	Range	Mean ± SD	Range	Mean ± SD	Range	Mean ± SD	Range
Feed Intake (kg DM/d)	4.06 ± 0.70	3.23 - 4.92	5.36 ± 0.71	4.74 - 6.38	6.24 ± 1.3	4.87 - 7.45	6.49 ± 0.89	5.24 - 7.32	6.54 ± 1.8	4.42 - 8.14
Initial liveweight (kg)	179 ± 38	133 - 225	181 ± 42	130 - 233	184 ± 35	149 - 221	179 ± 35	135 - 221	183 ± 54	117 - 235
Final liveweight (kg)	207 ± 34	167 - 249	255 ± 36	228 - 307	267 ± 50	222 - 327	265 ± 43	219 - 323	279 ± 76	191 - 347
Liveweight gain (kg/d)	0.091 ± 0.081	0.018 - 0.202	0.585 ± 0.095	0.470 - 0.702	0.792 ± 0.125	0.679 - 0.917	0.836 ± 0.095	0.714 – 0.940	0.943 ± 0.169	0.750 - 1.107
OM Digestibility (%)	54.3 ± 4.1	51.0 - 60.0	60.6 ± 2.1	57.7 - 62.7	62.8 ± 3.8	57.6 - 66.1	66.9 ± 2.3	64.5 - 70.0	75.1 ± 2.4	72.8 - 78.3

**Table 3:** Regression equations to estimate intake, digestibility, faecal N, and liveweight

	Regression equation ^1,2^	r^2^	Sig. of regression
Intake			
Concentrate intake (kg DM/d)	Y = −2.39 + 0.0117(I) + 2.68(C) - 0.348(C^2^)	0.97	<0.0001
Guinea grass intake (kg DM/d)	Y = 0.701 + 0.00782(I) - 0.321(C)	0.69	0.0002
Rice straw intake (kg DM/d)	Y = 0.747 + 0.00539(I) - 0.00808(C) - 0.209(C^2^)	0.93	<0.0001
Substitution rate (kg DM/kg DM)	Y = 1.16 - 0.00589(I) + 0.215(C)	0.85	<0.0001
^3^OM intake (kg/day)	Y = −0.996 + 0.0227(I) + 2.30(C) - 0.535(C^2^)	0.95	<0.0001
^4^GE intake (MJ/d)	Y = −18.9 + 0.438(I) + 43.8(C) - 10.1(C^2^)	0.94	<0.0001
^5^CP intake (kg/d)	Y = −0.278 + 0.00307(I) + 0.407(C) - 0.0693(C^2^)	0.97	<0.0001
^6^EE intake (kg/d)	Y = −0.114 + 0.000818(I) + 0.144(C) - 0.0221(C^2^)	0.97	<0.0001
^7^NDF intake (kg/d)	Y = 1.27 + 0.00886(I) - 0.240(C)	0.48	0.0036
Digestibility period			
OM digestibility (%)	Y = 54.4 + 8.00(C)	0.84	<0.0001
Digestible OM intake (kg/d)	Y = −0.149 + 0.0106(I) 0.971(C)	0.88	<0.0001
Gross energy digestibility (%)	Y = 75.0 + 13.6(C) - 4.36(C^2^)	0.43	0.0086
Digestible energy intake (MJ/d)	Y = −7.39 + 0.311(I) + 38.5(C) - 8.75(C^2^)	0.89	<0.0001
CP digestibility (%)	Y = 56.9 + 6.09(C)	0.63	<0.0001
EE digestibility (%)	Y = 9.28 + 0.0884(I) + 38.8(C) - 7.62(C^2^)	0.93	<0.0001
NDF digestibility (%)	Y = 59.8 - 8.06(C)	0.63	<0.0001
Faecal N (kg/d)	Y = −0.00168 + 0.000129(I) + 0.0203(C) - 0.00589(C^2^)	0.89	<0.0001
Liveweight			
Initial liveweight (kg)	n.a.	n.a.	0.9905
Final liveweight (kg)	Y = −13.7 + 1.12(I) + 64.9(C) - 15.6(C^2^)	0.97	<0.0001
Liveweight gain (kg/d)	Y = −163 + 1.43(I) + 773(C) - 185(C^2^)	0.90	<0.0001

^1^I: Initial body weight (kg)

^2^C: Amount of concentrate offered (% of liveweight)

^3^OM: Organic matter

^4^GE: Gross energy

^5^CP: Crude protein

^6^EE: Ether extract

^7^NDF: Neutral detergent fibre

**Table 4:** Least squares means, for the effect of different amounts of a concentrate mix on intake, digestibility, faecal N, and liveweight

	Concentrate Treatment (% of liveweight)											
	0		0.6		1.2		1.8		2.4		SE	Pr > F
Intake												
Concentrate intake (kg DM/d)	0.00	^a^	1.27	^b^	2.66	^c^	3.78	^d^	4.29	^d^	0.22	<0.0001
Guinea grass intake (kg DM/d)	2.19	^a^	2.17	^a^	1.94	^ab^	1.56	^b^	1.53	^b^	0.22	0.0198
Rice straw intake (kg DM/d)	1.83	^a^	1.72	^a^	1.56	^a^	1.11	^b^	0.61	^c^	0.11	<0.0001
Substitution rate (kg DM/kg DM)	n.a.		0.10	^a^	0.25	^ab^	0.37	^bc^	0.49	^c^	0.10	0.0143
^1^OM intake (kg/day)	3.57	^a^	4.63	^b^	5.59	^c^	5.93	^c^	5.96	^c^	0.25	<0.0001
^2^GE intake (MJ/d)	69.3	^a^	89.7	^b^	108.0	^c^	114.5	^c^	115.3	^c^	4.9	<0.0001
^3^CP intake (kg/d)	0.343	^a^	0.540	^b^	0.732	^c^	0.850	^d^	0.910	^d^	0.037	<0.0001
^4^EE intake (kg/d)	0.053	^a^	0.121	^b^	0.192	^c^	0.244	^d^	0.266	^d^	0.011	<0.0001
^5^NDF intake (kg/d)	2.97	^a^	3.01	^a^	2.86	^a^	2.38	^a^	2.56	^a^	0.33	0.2994
Digestibility period												
OM digestibility (%)	54.3	^a^	60.7	^b^	62.8	^bc^	66.9	^c^	75.1	^d^	2.2	<0.0001
Digestible OM intake (kg/d)	1.89	^a^	2.58	^b^	3.27	^c^	3.69	^cd^	4.25	^d^	0.27	<0.0001
Gross energy digestibility (%)	75.3	^c^	80.0	^bc^	87.8	^a^	83.3	^ab^	83.2	^ab^	3.1	0.0171
Digestible energy intake (MJ/d)	55.7	^a^	71.2	^b^	92.7	^c^	93.3	^c^	97.2	^c^	5.3	<0.0001
CP digestibility (%)	57.04	^ab^	62.1	^ab^	62.36	^ab^	66.6	^bc^	73.03	^c^	3.1	0.0019
EE digestibility (%)	25.5	^d^	51.4	^c^	59.7	^b^	71.8	^a^	76.7	^a^	3.9	<0.0001
NDF digestibility (%)	59.7	^a^	57.4	^a^	48.6	^b^	41.9	^b^	43.1	^b^	4.0	0.0012
Faecal N (kg/d)	0.025	^a^	0.0321	^b^	0.0413	^c^	0.0422	^c^	0.0386	^c^	0.0022	<0.0001
Liveweight												
Initial liveweight (kg)	199	^a^	206	^a^	200	^a^	195	^a^	200	^a^	31.4	0.9983
Final liveweight (kg)	208	^a^	248	^b^	266	^bc^	271	^c^	279	^c^	6.2	<0.0001
Liveweight gain (kg/d)	0.092	^a^	0.577	^b^	0.792	^c^	0.843	^c^	0.943	^c^	0.074	<0.0001

^a-d^ In each row, least squares means followed without a common superscript are significantly different (*P* < 0.05) according to Fisher’s LSD

^1^OM: Organic matter

^2^GE: Gross energy

^3^CP: Crude protein

^4^EE: Ether extract

^5^NDF: Neutral detergent fibre

### The effect of concentrate on intake

**Fig. 1:**
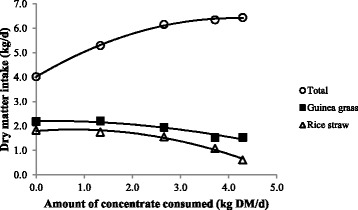
Effects of amount of concentrate consumed on total dry matter intake, rice straw intake, and Guinea grass intake. Values are averages of intakes (*n* = 4) measured across the whole experimental period

There was a significant (*p* < 0.0001) non-linear effect of treatments on concentrate intake (Table 3), with intake increasing from T0 to T3, but no difference in concentrate intake between T3 and T4 (Table 4). Guinea grass intake declined linearly with increasing concentrate offered (Table 3) and concentrate consumed (Fig. 1). Rice straw intake declined curvilinearly with increasing concentrate offered (Table 3) and concentrate consumed (Fig. 1).

Total dry matter intake increased curvilinearly from 4.0 to 6.4 kg/d as the amount of concentrate consumed increased (Fig. 1). The substitution rate of concentrate for Guinea grass and rice straw increased linearly with amount of supplement consumed (Table 3), and was as high as 0.49 kg DM/kg DM (Table 4).

**Fig. 2:**
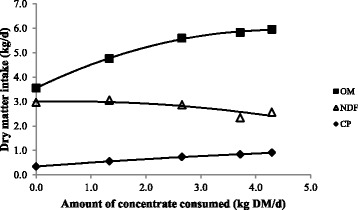
Effects of amount of concentrate consumed on organic matter intake, NDF intake, and CP intake. Values are averages of intakes (*n* = 4) measured across the whole experimental period

The intakes of OM, CP, EE, and GE, increased curvilinearly with increasing concentrate offered (Table 3); however there were no significant differences between the T3 and T4 treatments (Table 4). The ANOVA analysis indicates no significant effect of treatments on NDF intake (Table 4); however the regression analysis indicates a linear decline in NDF intake as the amount of concentrate consumed increased (Table 3). These results are reflected in Fig. 2 which shows concentrate consumed plotted against NDF, CP, and OM intake. Over the experimental range, as the level of concentrate intake increased, the OM, and CP intakes increased, however the NDF intake decreased.

### The effect of concentrate intake on liveweight gain

**Fig. 3:**
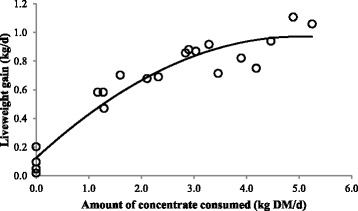
Effects of amount of concentrate consumed on liveweight gain of cattle fed a basal diet of Guinea grass and rice straw, measured across the whole experimental period. Values are for individual bulls

The mean weight gain of bulls ranged from 0.09 kg/d (T0) to 0.94 kg/d (T4) (Table 4). The concentrate treatments had a significant (*p* < 0.0001) effect on LWG (Table 3). There were significant differences in LWG between T0 to T1 to T2, but no difference in LWG between T2, T3 and T4 (Table 4). These results are reflected in Fig. 3, which shows a curvilinear relationship with LWG increasing as concentrate intake increases, but at a declining rate of increase.

### The effect of concentrate intake on digestibility

The digestibilities of dry matter, organic matter, crude protein, and crude fat increased with increasing concentrate level offered; however NDF digestibility decreased (Table 3). The faecal nitrogen content significantly (*P* < 0.0001) increased up until 1.2 % of LW, after which there was no increase with increasing amount of concentrate offered (Table 4).

## Discussion

The results support the hypothesis that total dry matter and organic matter intakes would increase in a curvilinear response as the amount of the formulated concentrate offered increased up to 2.4 % LW. There was no significant difference in concentrate intake between treatments containing concentrate levels of 1.8 and 2.4 % LW. Roughage intake declined with increasing intake of concentrate. This result is consistent with previously published reports where supplements have been fed to provide energy and/or protein to cattle consuming low quality forages [9, 13, 14]. Intake of rice straw or basal forage diets declines as the amount of concentrate containing cassava powder consumed increases [2, 15, 16]. If the amount of fermentable carbohydrate in cattle diet is higher than 15 % of total dry matter intake, roughage intake decreases [17].

There are many factors that affect the total dry matter and roughage intakes in ruminants, including diet quality and feeding management. In our study, there was no increase in Guinea grass intake at the lowest level of concentrate supplementation, because all of the offered grass was consumed. The positive effects of small amounts of supplement on intake of low and medium quality forages have been reported elsewhere [14, 18, 19]. However, with increasing concentrate, substitution invariably occurs [20] and increases as the amount of concentrate consumed increases [2].

The decline in NDF digestibility with increasing concentrate consumption is consistent with reports by Ba et al. [2, 3] and Dung et al. [21]. There is evidence to suggest that the digestibility of NDF in mature forages may be depressed more than that of fresh herbages when the rumen environment is altered by feeding concentrates [22, 23]. Dixon and Stockdale [24] suggest that reduced NDF digestion is a primary cause of substitution. Many studies have concluded that increased concentrate intake contributes to a reduction of rumen pH and cellulolytic bacterial activity, which decreases the digestion of fibre [25–27]. It was not feasible to estimate the digestibility of different dietary ingredients in this experiment. However, if the digestibility of concentrate NDF remained constant across T1 to T4, then the digestibility of Guinea grass and/or rice straw NDF must have declined markedly as the amount of concentrate consumed increased. This indicates that the amount of metabolisable energy derived from Guinea grass and rice straw declined due to substitution and negative associative effects on their NDF digestibility as the amount of concentrate consumed increased.

The present results support the hypothesis that LWG increases curvilinearly with increasing amounts of concentrate, and that a maximum level of LWG is reached. This relationship is described by the following equation:

$$ \mathrm{L}\mathrm{W}\ \mathrm{gain}\ \left(\mathrm{kg}/\mathrm{day}\right) = 163 + 1.43\left(\mathrm{I}\right) + 773\left(\mathrm{C}\right) - 185\ \left({\mathrm{C}}^2\right)\ \left({\mathrm{R}}^2 = 0.90;\ \mathrm{p}<0.0001\right) $$

where I indicates the initial body weight (kg) and C indicates the level of concentrate treatment (% of LW). The equation does not indicate the optimum economic level of supplementation, which needs to also take into account purchased input prices and selling price.

The improved LWG for these experiments is likely due to the increased DM intake, OM intake and OM digestibility resulting from increased intake of concentrate. The results are similar to those of previous experiments. A number of studies reported that LWG increased linearly as the concentrate intake increased [2, 21, 28, 29]. We purposefully included high amounts of concentrate supplementation to show that the linear relationship would not hold across a wide range in amounts of supplement offered or consumed and that there are diminishing responses at high amounts of supplementation. This has important consequences in terms of the profit derived from supplementary feeding.

## Conclusions

This experiment examined the effects of supplementing young Vietnamese Brahman-cross growing cattle with a concentrate mix based on ingredients locally available in Central Vietnam. Liveweight gain increased with increasing the level of supplementation up to 1.2 % of LW; however there was minimal change in LW with increasing amounts of supplementation beyond 1.2 %. An equation for estimating LWG based on the amount of supplementation was developed, and could be used for determining the optimal supplementation strategy if combined with information on input costs and cattle sale prices.

## Abbreviations

CP: Crude protein
DM: Dry matter
EE: Ether extract
GE: Gross energy
LW: Liveweight
LWG: Liveweight gain
NDF: Neutral detergent fibre
N: Nitrogen
OM: Organic matter
